# British Madeira

**Published:** 1831

**Authors:** 


### British Madeira.

We do not mean the wine which is manufactured in this country, and passed off as the produce of Madeira; but a Winter residence on our own shores, combining many of the good qualities of certain celebrated foreign climes with the comforts of an English fireside. The situation to which we allude is Undercliff, in the Isle of Wight, which has been recognised by medical men as no mean substitute for Madeira, Nice, Pisa, and other fashionable places of resort for British invalids during the gloomy skies of an English Winter. We have reason to believe that speculation is afloat in providing comfortable accommodations for those who may be disposed to try the effects of the above-mentioned sheltered and salubrious spot, in preference to the hazardous journey to Italy or the South of France. We have just received the following notice.

"Mount Cleeves House, Undercliff, Niton, Isle of Wight, distant one mile from the Sand-rock Chalybeate Spring.

This house, with its lawns and pleasure-grounds and dry walks reaching to the foot of the Upper Cliff, extending over ten acres of land, is opened for the reception of select Family Parties. Attached to it is a Bathing-house and Dressing-room on the sea-shore immediately below, a luxury not elsewhere to be easily obtained; and a Dairy is kept for the supply of milk and butter.

The peculiarly dry and healthy situation of the House and Grounds; its proximity to the Sand-rock Spring, distant one mile, a favourite walk; the extreme purity of the water and convenience for sea-water bathing,—render Mount Cleeves a most desirable residence for persons in delicate health. The mildness of its climate is proverbial, fitting it for a Winter's residence for invalids, scarcely inferior to the island of Madeira, whilst it is much superior to it in the advantages which it offers in point of convenience of distance for removal and medical attendance.

There is a daily delivery of Letters and Newspapers at the House. The best route is by Portsmouth, where the Ryde Steam-Packets await the arrival of the London Day Coaches: the Packets are met at Ryde by the Newport Coaches. At Newport vehicles of every description may be obtained. By this route passengers from London may reach Mount Cleeves at Eight or Nine o'clock in the evening, or in about 12 hours.

Small parties, wishing to be relieved from the trouble of providing for themselves, may be accommodated with partial Board at Mrs. Pedder's table.

For Terms and other Particulars apply [Letters to be post-paid] to Mr. Pedder, at Mount Cleeves House, Under-Cliff, Isle of Wight.

References may be made to Mr. Hunter, Bookseller, 72, St. Paul's Church-yard, London."

The very unsettled state of the Continent, and the knowledge which Englishmen have dearly obtained of the inutility, not to say injury, in many instances and in many complaints, of an Italian climate, will prove a great check to the emigration of invalids for some time to come. It is, therefore, of some consequence to know that, at Under-Cliff, commodious quarters may be obtained for invalids, with the advantage of a climate scarcely inferior to that of Madeira.

---

XXVI.

